# Supply and demand for antimalarial drugs and diagnostic testing in the era of subsidies: multi-country findings

**DOI:** 10.1186/1475-2875-11-S1-O15

**Published:** 2012-10-15

**Authors:** Tanya Shewchuk

**Affiliations:** 1 ACTwatch PSI, Nairobi, Kenya

## 

Abstract not submitted

